# *Moringa oleifera* L. leaf extract attenuates neuroinflammation and behavioral alterations in a fibromyalgia mice model: Modulation of serotonin and cytokine pathways

**DOI:** 10.1016/j.ibneur.2026.02.010

**Published:** 2026-02-15

**Authors:** Ohoud H. Alhawiti, Ashwaq H. Batawi, Mona A. AL-Thepyani, Reham Tash, Asma Almuhammadi, Ashwaq Hassan Alsabban, Sheren A. Azhari, Badrah S. Alghamdi

**Affiliations:** a Department of Biological Science, Faculty of Science, King Abdulaziz University, P.O.Box 80200, Jeddah 21589, Saudi Arabia; b Department of Chemistry, College of Sciences & Arts, King Abdulaziz University, Rabigh 21911, Saudi Arabia; c Neuroscience and Geroscience Research Unit, King Fahd Medical Research Center, King Abdulaziz University, Jeddah 21589, Saudi Arabia; d Faculty of Medicine, King Abdulaziz University, Anatomy and Embryology Department, Rabigh 25724, Saudi Arabia; e Faculty of Medicine, Ain Shams University, Anatomy and Embryology Department, Abassia, Cairo 11566, Egypt; f King Abdulaziz University, Rabigh 25724, Saudi Arabia; g Unit of Neurological Disorders, Faculty of Medicine, Princess Al-Jawhara Center of Excellence in Research of Hereditary Disorders (PACER.HD), Rabigh 25724, Saudi Arabia; h Clinical Physiology Department, Faculty of Medicine, King Abdulaziz University, Saudi Arabia

**Keywords:** Reserpine, Fibromyalgia, Behavioral symptoms, Cytokines, Neurotransmitters, *Moringa oleifera*

## Abstract

**Background:**

Fibromyalgia (FM) is a complex, chronic disorder characterized by widespread pain, fatigue, cognitive impairment, and sleep disturbances, and it is considered the second most prevalent rheumatic condition. Current pharmacological therapies are often associated with undesirable side effects, underscoring the need for safer, natural therapeutic alternatives. *Moringa oleifera* leaves are rich in nutrients and bioactive antioxidants and have demonstrated therapeutic potential in inflammatory and immune-related disorders.

**Methods:**

This study investigated the therapeutic effects of *M. oleifera* leaf extract in a reserpine-induced fibromyalgia model using multiple treatment groups of male mice. Behavioral, histological, and neurochemical assessments were conducted.

**Results:**

Mice with reserpine-induced FM exhibited reduced body weight, decreased paw withdrawal threshold and thermal latency, diminished locomotor activity and grooming behavior, impaired spontaneous alternation, and prolonged immobility time. Histopathological examination revealed marked structural disruption of hippocampal tissue, accompanied by reduced serotonin levels and elevated concentrations of IL-1β, TNF-α, and NO compared with control animals. Treatment with *M. oleifera* significantly attenuated these alterations by improving behavioral performance, restoring hippocampal architecture, and normalizing serotonin levels and pro-inflammatory markers.

**Conclusions:**

These findings indicate that *M. oleifera* exerts a protective effect against reserpine-induced fibromyalgia, likely through modulation of serotonergic activity and suppression of inflammatory cytokine signaling pathways.

## Graphical Abstract

**Figure:**
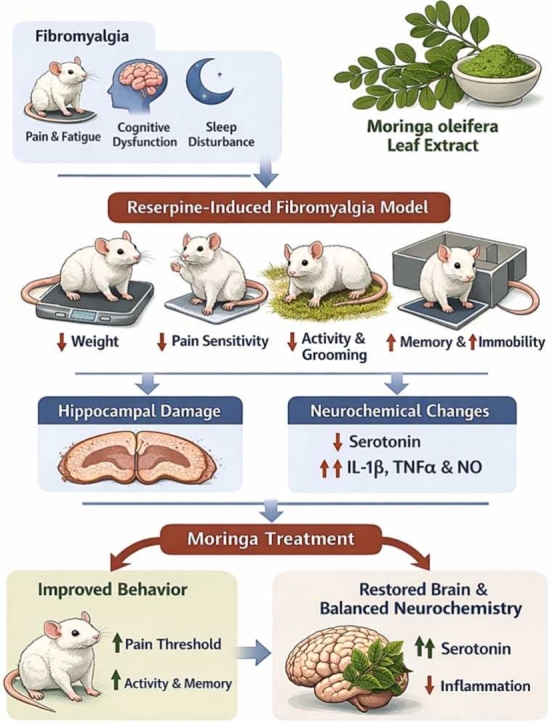

## 1 Introduction

Fibromyalgia (FM) is a multifactorial and complex neurological syndrome characterized by chronic, widespread musculoskeletal pain accompanied by a constellation of symptoms including fatigue, sleep disturbances, cognitive impairment, depression, and headaches (Singh et al., 2021). The condition is often challenging to diagnose due to its overlapping symptoms with other chronic pain syndromes and the absence of specific diagnostic biomarkers (Giorgi et al., 2022). Despite decades of research, the exact etiology and pathophysiological mechanisms underlying FM remain incompletely understood, which has complicated the development of effective therapeutic interventions.

Epidemiologically, FM affects approximately 5 % of the global population, predominantly occurring between the ages of 20 and 60 years (Gilheaney and Chadwick, 2024). A recent epidemiological survey in the Kingdom of Saudi Arabia reported a notably high prevalence of FM, particularly among women, with rates reaching 13.4 % (Bawazir, 2023). This increased incidence highlights the necessity for enhanced public health awareness, early detection strategies, and educational programs targeting healthcare professionals and the general population in Saudi Arabia (Althobaiti et al., 2022).

At the pathophysiological level, FM is primarily associated with central nervous system (CNS) dysfunction involving altered pain perception and abnormal neurotransmission within nociceptive and antinociceptive pathways (Yao et al., 2020). Dysregulation of monoaminergic neurotransmitters, especially serotonin, dopamine, and norepinephrine, plays a critical role in the amplification of pain signals. Elevated levels of excitatory neurotransmitters, such as glutamate and substance P, coupled with decreased serotonergic activity, contribute to central sensitization and hyperalgesia in affected individuals (Rus et al., 2024, Dumolard et al., 2023). Moreover, growing evidence implicates oxidative stress, neuroinflammation, neuroendocrine imbalances, genetic predisposition, environmental exposures, and psychosocial stressors as contributing factors in the onset and progression of FM (Vincent et al., 2013).

Currently, there is no definitive cure for FM. Symptomatic management often relies on pharmacological agents including antidepressants, anticonvulsants, sleep aids, and analgesics such as opioids (Vincent et al., 2013). However, the long-term use of these drugs is frequently limited by their modest efficacy and significant adverse effects, including hepatotoxicity and dependency (de la Cruz Cazorla, et al., 2024). Consequently, there has been an increasing shift toward exploring safer and more effective alternative therapies, particularly those derived from natural sources. Wang et al. (2018) emphasized that many conventional drugs used in FM management provide only transient relief, underscoring the need for novel therapeutic options with improved safety profiles. Medicinal plants have garnered particular interest as they contain diverse bioactive compounds capable of modulating oxidative stress, inflammation, and neurotransmitter balance, thereby offering potential benefits for FM management (Gómez-Centenoet al., 2022).

Among various medicinal plants, *M. oleifera* has attracted considerable attention due to its broad pharmacological potential. This plant, commonly known as the miracle tree, is rich in essential nutrients, including minerals, vitamins, beta-carotene, proteins, and potent antioxidants (Al-Abri et al., 2018, Yang et al., 2020). Traditionally, *M. oleifera* has been utilized in many developing regions to combat malnutrition and as a therapeutic remedy for conditions such as malaria, typhoid, and arthritis (Stohs and Hartman, 2015). Phytochemical analyses have revealed that *M. oleifera* leaves contain a wide range of bioactive constituents, including flavonoids, phenolics, anthraquinones, saponins, and phenolic acids such as caffeic and chlorogenic acids, as well as flavonoids like quercetin, isorhamnetin, and apigenin, all known for their antioxidant, anti-inflammatory, and neuroprotective activities (Khan et al., 2021).

Given the crucial role of oxidative stress and inflammation in FM pathogenesis, the bioactive compounds present in *M. oleifera* may exert protective and restorative effects on neural tissues. Therefore, the present study aimed to evaluate the potential therapeutic efficacy of *M. oleifera* leaf extract in a reserpine-induced fibromyalgia model in male mice, with a comprehensive assessment encompassing behavioral, biochemical, and histopathological parameters.

## 2 Methods

### 2.1 Chemicals and reagents

Gallic acid: Stock solution of 2 mg/mL (Catalogue No. AC410860050, Thermo Scientific, USA) in methanol (Catalogue No. A452–4, Thermo Scientific, USA) was prepared, from which working dilutions of 1000, 750, 500, 375, and 250 µg/mL were performed. Acetic acid glacial (Catalogue No.20104.334, VWR, France). Reserpine: Administered at a dose of 0.5 mg/kg (Catalogue No.132280050, 98 % purity, Acros Organics -Fisher Scientific, USA). Serotonin: Measured using mouse ELISA kits (Catalogue No. SEKSM0016) supplied by Solarbio Science & Technology Co., Ltd., Beijing, China. Interleukin-1β (IL-1β) and Tumor Necrosis Factor-alpha (TNF-α): Measured using mouse ELISA kits (Catalogue No. SEKM-0002 and SEKM-0034, respectively) supplied by Solarbio Science & Technology Co., Ltd., Beijing, China. Nitric Oxide (NO): Measured using mouse ELISA kits (Catalogue No. E0439Mo) supplied by BT LAB Bioassay Technology Laboratory, Zhejiang, China, following the manufacturer’s instructions.

### 2.2 Animals

A total of 80 male Swiss albino mice (30–43 g, 9–10 weeks old) were obtained from the King Fahd Medical Research Center (Jeddah, Saudi Arabia). The animals were randomly divided into experimental groups (20 mice per group) and housed in transparent acrylic cages containing dust-free sawdust bedding under controlled conditions (temperature 23 ± 2 °C, humidity 55 ± 10 %, and a 12:12 h light–dark cycle). Mice were acclimatized for five days prior to experimentation. All procedures were conducted in accordance with the guidelines of the Animal Care and Use Committee (ACUC) of King Fahd Medical Research Center, and the study protocol was approved by the Biomedical Ethics Research Committee of King Abdulaziz University (Ethical Approval No. 358–24).

### 2.3 Model of experimental fibromyalgia induced by reserpine

Experimental fibromyalgia (FM) was induced in mice using reserpine (Thermo Fisher Scientific, USA) following the protocol described by AboTaleb et al. (AboTaleb et al., 2024). Reserpine was dissolved in 0.5 % glacial acetic acid diluted with distilled water (v/v) and administered subcutaneously at a dose of 0.5 mg/kg once daily for three consecutive days.

### 2.4 Plant material and preparation of methanolic leaves extraction

Fresh *M. oleifera* L. leaves were obtained from the Botanic Garden of the Faculty of Science, Alexandria University, Egypt. The plant material was taxonomically identified and authenticated by Prof. Amal M. Fakhary (Plant Ecology and Biodiversity, Faculty of Science, Alexandria University). A voucher specimen (No. 4110) was deposited in the Alexandria University Herbarium (ALEX).

Methanolic *Moringa oleifera* leaf extract (MMOLE) was prepared as previously described with minor modifications (Alghazeer et al., 2012). Fresh *M. oleifera* leaves were air-dried at room temperature for two weeks and subsequently pulverized using an electric blender. Approximately 500 g of the powdered material was macerated in 70 % methanol for 72 h with intermittent agitation. The resulting mixture was filtered through muslin cloth to separate the filtrate from the plant residue. The filtrate was transferred to a round-bottom flask and subjected to heating at 60 °C for 1 h using a Soxhlet apparatus. Thereafter, the extract was concentrated in a water bath at 100 °C for 24 h to remove the solvent. The final extract yield was 11.72 % (w/w).

### 2.5 High-performance liquid chromatography (HPLC) analysis

The total phenolic content of the *M. oleifera* leaf extract was determined using the Folin–Ciocalteu method as described by Kiranmai et al. (2011), with the resulting blue complex measured at 630 nm. Total flavonoid content was assessed using the aluminum chloride colorimetric method according to Kiranmai et al. (2011), with the resulting yellow complex measured at 420 nm.

### 2.6 Experiment design

**Fig. 1:**
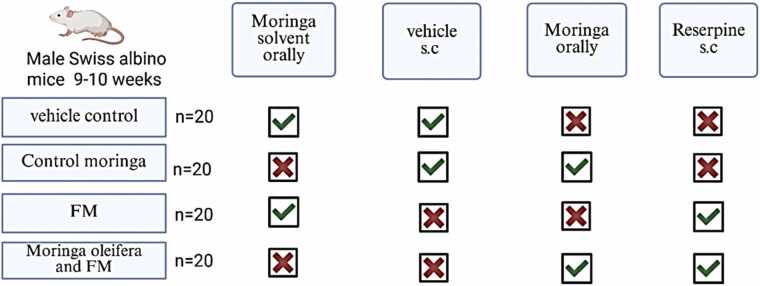
Experimental group design (Created with BioRender.com).

**Fig. 2:**
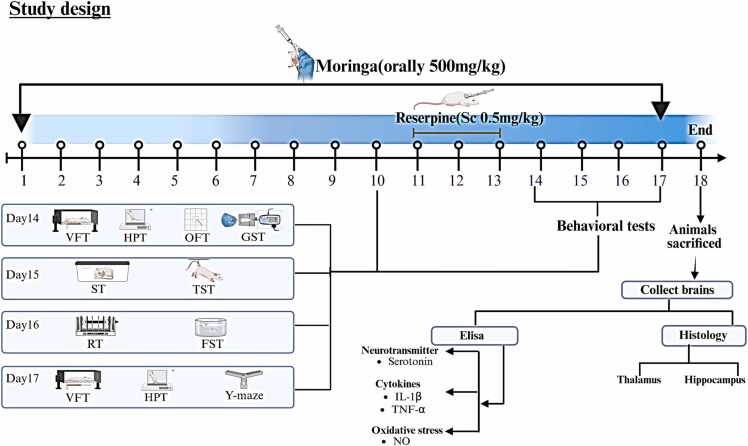
Experimental timeline. VFT: Von Frey Test, HPT: Hot Plate Test, OFT: Open Field Test, GST: Grip Strength Test, ST: Splash Test, TST: Tail Suspension Test, RT: Retention time, FST: Forced Swimming Test, IL-1β: Interleukin-1 Beta, TNF-α: Tumor Necrosis Factor alpha, NO: Nitric Oxide (Created with BioRender.com).

A total of 80 mice were randomly assigned into four groups (n = 20 each) as follows: Group I (Control): Received distilled water orally from day 1–17. From day 11–13, mice were injected subcutaneously with 0.5 % glacial acetic acid in distilled water, 3 h after water administration. From day 14–17, only distilled water was administered. Group II (*M. oleifera*): Received *M. oleifera* leaf extract (500 mg/kg body weight, orally) from day 1–17. From day 11–13, mice were injected subcutaneously with 0.5 % glacial acetic acid, 3 h after extract administration. From day 14–17, mice continued to receive the extract only (Aljazzaf et al., 2023). Group III (Reserpine): Received distilled water orally from day 1–17. From day 11–13, mice were injected subcutaneously with reserpine (0.5 mg/kg body weight), 3 h after water administration. From day 14–17, only distilled water was administered (Kaur et al., 2020). Group IV (*M. oleifera* + Reserpine): Received *M. oleifera* extract (500 mg/kg body weight, orally) from day 1–17. From day 11–13, mice were injected subcutaneously with reserpine (0.5 mg/kg body weight), 3 h after extract administration. From day 14–17, mice continued to receive the extract only. Behavioral assessments evaluating sensory response, depressive-like behavior, and motor activity were performed from day 14–17 (AboTaleb et al., 2024). On day 18, mice were sacrificed, and brain tissues were collected for further analyses. The experimental timeline and design are summarized in Fig. 1, Fig. 2.

### 2.7 Body weight measurement

Body weights of all mice were recorded daily from day 1 to day 18 to assess the effects of reserpine and *M. oleifera* extract. Percentage weight change was calculated using the formula (Alqurashi et al., 2022):

Change in body weight (%) = [(Day weight - initial weight)/ initial weight]x100.

### 2.8 Behavioral study

#### 2.8.1 Evaluation of depression - like behavior

The following behavioral tests were selected to reflect specific FM-like domains: mechanical and thermal nociceptive tests assessed widespread pain sensitivity; open-field activity and grooming behavior reflected fatigue and motivational deficits; spontaneous alternation evaluated cognitive dysfunction (“fibro-fog”); and immobility time assessed depressive-like behavior, which is highly prevalent in FM patients (Sluka and Clauw, 2016).

##### 2.8.1.1 Forced swimming test (FST)

The forced swimming test (FST) was conducted to assess depression-like behavior and evaluate the potential antidepressant effects of treatments (Toledano-Martos et al., 2024). On day 16, each mouse was placed individually in a transparent cylindrical container (10 cm diameter, 20 cm height) filled with water (14 cm depth, 25 ± 1 °C) for 6 min. The duration of immobility was recorded during the last 4 min of the test. Immobility was defined as the absence of active movements except those necessary to maintain the animal’s head above water.

##### 2.8.1.2 Splash test (ST) - Anti-anhedonia-like behavior

The splash test was conducted on day 15 to assess anhedonia-like behavior in mice (Martins et al., 2022). Each mouse received 0.3 mL of a 10 % sucrose solution sprayed onto the dorsal fur, and grooming behavior was recorded for 5 min. Reduced grooming duration was considered indicative of anhedonia or depression-like behavior, and was negatively correlated with immobility time in the FST.

##### 2.8.1.3 Tail suspension test (TST)

The TST was used to evaluate depression-like behavior in mice (Cryan et al., 2005). On day 15, each mouse was suspended by the tail (approximately 2 cm from the tip) using adhesive tape affixed to a 50 cm-high board, with visual isolation from other animals. The total duration of immobility was recorded during the last 4 min of a 6 min test session. Prolonged immobility time was interpreted as an indicator of depression-like behavior.

#### 2.8.2 Pain behavior assessment

##### 2.8.2.1 Von Frey test (VFT)

Mechanical allodynia was assessed using the von Frey ascending–descending method as described by Gonzalez-Cano et al. (2018) on days 14 and 17. Each mouse was placed individually in a transparent acrylic chamber (90 × 38 cm) with a wire mesh floor and allowed to acclimate for 45 min. Calibrated von Frey filaments (0.04–4 g), starting at 0.6 g, were applied perpendicularly to the plantar surface of the hind paw until slight bending occurred. A positive response was defined as rapid paw withdrawal, licking, or shaking. The paw withdrawal threshold (g) was calculated using the up–down method according to Christensen et al. (2020). A reduction in withdrawal threshold was interpreted as increased mechanical sensitivity.

##### 2.8.2.2 Hot plate test (HPT)

Thermal nociceptive thresholds were assessed using a hot plate apparatus (Ugo Basile, Italy) following the method of Hunskaar et al. (1986) on days 14 and 17. Mice were placed individually on a metal surface maintained at 55 °C, and the latency to a pain response, indicated by hind paw licking or jumping, was recorded. A cutoff time of 30 sec was applied to prevent tissue injury. To minimize habituation effects, each group was subdivided and tested twice under controlled conditions (Deuis et al., 2017).

#### 2.8.3 Memory assessment

##### 2.8.3.1 Y-maze test

Spatial short-term memory was evaluated on day 17 using the Y-maze test as described by Hughes (2004). The maze consisted of three arms (A, B, and C), each 10 cm wide and 15 cm high. Mice were placed individually in the central area and allowed to explore freely for 8 min in a quiet environment. The sequence of arm entries was recorded, and spontaneous alternation was defined as consecutive entries into three different arms (e.g., ABC, BCA). The percentage of spontaneous alternation was calculated as:

Spontaneous Alternation Percentage (%) = [(Number of Alternations)\(Total Number of Entries)- 2]× 100.

The percentage of spontaneous alternation was used as an index of spatial short-term memory performance.

#### 2.8.4 Evaluation of motor activity

##### 2.8.4.1 Rotarod test

Motor coordination and balance were evaluated using the rotarod test on day 16 (Altarifi et al., 2019). Mice were first trained to remain on a rotating rod for 30 s at a constant speed, followed by a 1 h rest period. For testing, each mouse was placed on the rod rotating at 14 rpm, and the latency to fall was recorded, with a maximum cutoff time of 240 sec. Three trials were conducted per mouse with 10 min intervals, and the mean latency to fall was calculated.

##### 2.8.4.2 Grip Strength test (GST)

Muscle strength was assessed on day 14 using a grip strength meter (Columbus Instruments, Columbus, OH, USA) (Brusco et al., 2021). Each mouse was allowed to grasp a metal grid with its forepaws, after which it was gently pulled by the tail in a horizontal direction until it released the grid. The maximal force) in grams of force (gf) was automatically recorded by the device. Each mouse performed three trials with 1 min intervals, and the mean value was calculated.

##### 2.8.4.3 Open field test (OFT)

Locomotor activity was assessed on day 14 by measuring total distance moved (TDM) and movement speed (Alzahrani et al., 2022). Each mouse was placed individually in a transparent square arena (45 × 45 × 34 cm) for 3 min under low lighting in a quiet environment to minimize stress. Activity was recorded and analyzed using the EthoVision XT8A tracking system (Noldus Information Technology, Wageningen, The Netherlands).

### 2.9 Histopathological procedures

*Brain sampling*: At the end of the experimental period (day 18), all mice were anesthetized and euthanized by cervical dislocation. Brains from each group (n = 20) were carefully excised. Ten brains per group were washed with normal saline and fixed in 10 % neutral buffered formalin for histopathological evaluation, while the remaining ten were stored at −80°C for biochemical analyses.

Histopathological examination was performed according to the method described by Alqurashi et al. (2022). Brain tissues were sectioned coronally and sagittally, placed in tissue cassettes, and processed using a tissue processor (Tissue-Tek® VIP™ 5 Jr-E2, Sakura, Japan) for 15 h. The tissues were embedded in paraffin, and serial sections of 3 μm thickness were obtained using a microtome (LEICA RM 2125 RTS, Leica Biosystems, China). Sections were mounted on glass slides and stained with hematoxylin and eosin (H&E) using an automated staining system (Automatic Stainer AUS240 Plus, Bio-Optica, Italy). Microscopic examination of the hippocampal (dentate gyrus region) and thalamic areas was performed at magnifications of × 100, × 200, × 400, × 600, and × 1000 using an Olympus BX51 light microscope (Olympus Corporation, Tokyo, Japan).

### 2.10 Biochemical analysis

#### 2.10.1 Neurotransmitter analysis

Serotonin levels were measured in brain tissues using a mouse ELISA kit (SEKSM-0016, Solarbio, Beijing, China) following the manufacturer’s protocol. Briefly, 50 μL of samples and standards were added to each well, followed by 50 μL of biotin-conjugated anti-5-HT antibody and incubated at 37 °C for 45 min. After washing, 100 μL of streptavidin-HRP was added and incubated for 30 min at 37 °C. The reaction was developed with 90 μL substrate solution and stopped after 30 min with 50 μL stop solution. Absorbance was measured at 450 nm using a microplate reader (BioTek Instruments, Winooski, VT, USA).

#### 2.10.2 Measurement of cytokines

Levels of interleukin-1β (IL-1β) and tumor necrosis factor-alpha (TNF-α) were quantified in brain tissues using mouse ELISA kits (SEKM-0002 and SEKM-0034, Solarbio, Beijing, China) following the manufacturer’s instructions. Briefly, 100 μL of homogenized samples and standards were added to microplate wells and incubated at 37 °C for 90 min. After washing, 100 μL of biotin-conjugated anti-IL-1β or anti-TNF-α antibodies was added and incubated for 60 min at 37 °C. The reaction was developed with 100 μL substrate solution for 15 min, stopped with 50 μL stop solution, and absorbance was read at 450 nm using a microplate reader (BioTek Instruments, Winooski, VT, USA).

#### 2.10.3 Measurement of oxidative stress

Nitric oxide levels were measured in brain tissues using a mouse ELISA kit (E0439Mo, BT LAB Bioanalytical Technology, Zhejiang, China) following the manufacturer’s protocol. Briefly, 40 μL of homogenized samples and 50 μL of standards were added to microplate wells, followed by 10 μL of anti-NO antibody and 50 μL of streptavidin-HRP. After incubation at 37 °C for 60 min, wells were washed, and 50 μL each of substrate solutions A and B were added and incubated for 10 min at 37 °C in the dark. The reaction was stopped with 50 μL stop solution, and absorbance was measured at 450 nm using a microplate reader (BioTek Instruments, Winooski, VT, USA).

### 2.11 Statistical analysis

Data were analyzed using GraphPad Prism software (version 10, GraphPad Software, San Diego, CA, USA). One- or two-way ANOVA followed by Tukey’s post hoc test was performed to compare differences among groups. Results are expressed as Mean±SEM, and statistical significance was set at *p* *<* 0.05. Individual data points were provided into each bar figures according to (Ghotbeddin et al., 2024; Mohammadkhani e al., 2024; Popovičová et al., 2024; Singsai et al., 2024)

## 3 Results

### 3.1 HPLC analysis of the *M. Oleifera* methanolic leaves extract

**Fig. 3:**
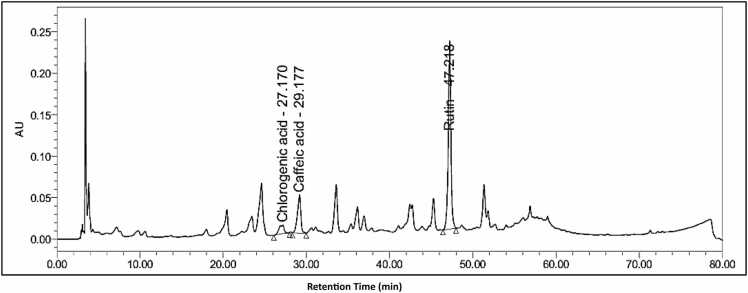
The spectrum of active compounds (Chlorogenic acid, Caffeic acid and Rutin) of *M. oleifera* leaves extract identified by HPLC analysis.

HPLC-based quantitative analysis of *M. oleifera* extract identified three major phytoconstituents: caffeic acid, chlorogenic acid, and rutin (Fig. 3). As shown in Table 1, rutin a flavonoid compound exhibited the highest retention time (RT), peak area, and concentration, followed by the phenolic compounds caffeic acid and chlorogenic acid. The concentrations of these compounds were 16.33 mg/g for rutin, 0.89 mg/g for caffeic acid, and 0.53 mg/g for chlorogenic acid in *M. oleifera* extract.

**Table 1:** Show phytochemicals screening of *M. oleifera* L. extract by using HPLC.

**Peak Name**	RT*	**Area**	**% Area**	**Height**
Chlorogenic acid	27.170	453395	6.70	10581
Caffeic acid	29.177	1279889	18.91	46125
Rutin	47.218	5033825	74.39	226548

*R.T. Retention time

### 3.2 The effect of *M. oleifera* on mice weight and short-term spatial memory

**Fig. 4:**
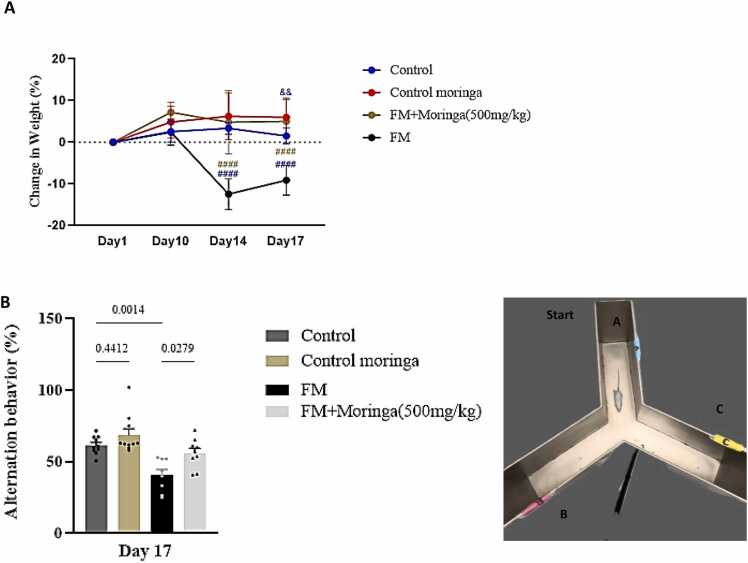
The Protective effect of *M. oleifera* on the body weight (A), short**-**term memory by Y- maze test (B) in FM mice model induced by reserpine (Schematic representation of the Y-maze test: Spontaneous alternation behavior is determined by successive entries into all three arms (e.g., ABC, BCA, or CAB), whereas repeated visits to the same arm within a sequence (e.g., ABA, CAC, or BCB) are considered errors). The data are presented as vertical lines indicate the Mean±SEM for each group (n = 20 mice), (&,&:(*p <* 0.01), #,#,#,#:(*p* *<* 0.001) indicate a significant difference between groups using two-way repeated-measures ANOVA, followed by Tukey’s multiple comparisons test.

Administration of *M. oleifera* extract showed no significant change in body weight compared with the control group on days 10 and 14. However, a significant increase (*p* *<* 0.05) was observed on day 17 (Fig. 4A). In contrast, the FM group showed no difference from controls on day 10 but exhibited a significant reduction in body weight on days 14 and 17(*p* *<* 0.0001, and *p* *<* 0.0001, respectively). Notably, mice in the FM + *M. oleifera* group displayed a significant weight gain compared with the FM group on days 14 and 17 (*p* *=* 0.0037). On the other hand, the Y-maze test on day 17 showed no significant difference in spontaneous alternation percentage between the control and *M. oleifera* groups (*p* *=* 0.4412) (Fig. 4B). In contrast, reserpine significantly reduced spontaneous alternation compared to the control (*p* *=* 0.0014). The FM + *M. oleifera* group demonstrated improved short-term spatial memory, evidenced by increased spontaneous alternation relative to the reserpine group (*p* *=* 0.0279) (Fig. 4B).

### 3.3 Effect of *M. oleifera* on mechanical and thermal hypersensitivity

**Fig. 5:**
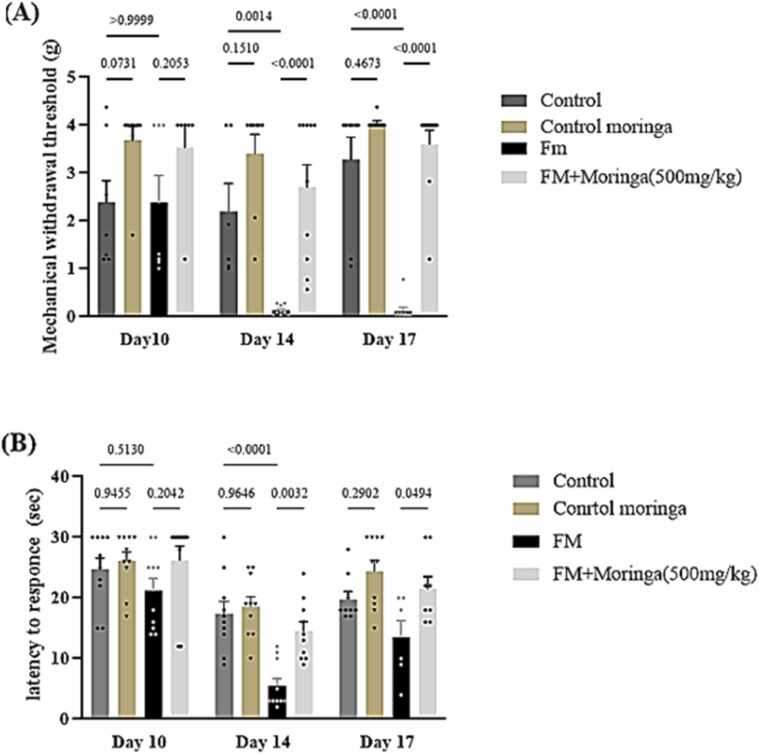
The protective effect of *M. oleifera* on mechanical hypersensitivity and thermal threshold in FM mice model. (A) Von Frey test to evaluate mechanical withdrawal threshold. **(B)** Hot plate to evaluate latency to response. The data are presented as vertical lines indicate the Mean±SEM for each group (n = 20 mice), Exact *p*-values are shown above each column to indicate a significant difference between groups. used two-way repeated-measures ANOVA, followed by Tukey’s multiple comparisons test.

The effect of *M. oleifera* on pain behavior in the FM mouse model was evaluated using the von Frey and hot-plate tests (Fig. 5). In the von Frey test, no significant differences were observed among groups on day 10 (Fig. 5A). Mice treated with *M. oleifera* alone showed no significant change compared with controls on days 14 and 17 (*p =* 0.1510 and *p =* 0.4673, respectively). Reserpine administration significantly reduced the paw withdrawal threshold on days 14 (*p =* 0.0014) and 17 (*p <* 0.0001). Conversely, *M. oleifera* treatment markedly increased the pain threshold in the FM+ *M. oleifera* group, as mice showed reduced sensitivity to pressure compared with the FM group on both days 14 and 17 (*p <* 0.0001 for both).

In the hot-plate test, *M. oleifera* administration showed no significant difference from the control group on days 14 and 17 (*p =* 0.9657 and *p* *=* 0.1677, respectively) (Fig. 5B). Reserpine-treated mice exhibited a significant reduction in latency time, indicating increased thermal pain sensitivity on days 14 (*p* *<* 0.0001) and 17 compared with controls. Conversely, *M. oleifera* treatment significantly increased latency times in the FM + *M. oleifera* group, confirming its antinociceptive effect, as mice required more time to respond to heat on both test days (*p* *=* 0.0032 and *p =* 0.0494, respectively).

### 3.4 The protective effect of *M. oleifera* on motor activity in FM mice model

**Fig. 6:**
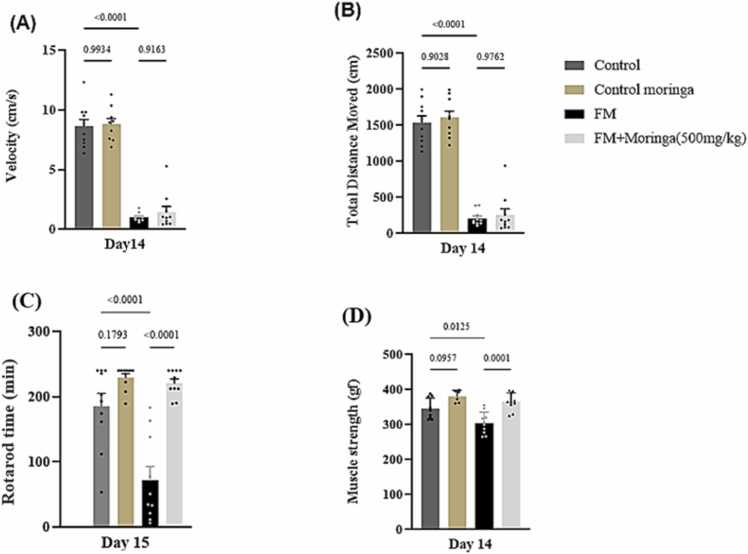
The protective effect of *M. oleifera* on (A) velocity, (B) TDM, (C) rotarod time and (D) grip strength in FM mice model induced by reserpine. SEM present by the vertical lines for each group (n = 20 mice), and all bars show mean. Exact *p*-values are shown above each column to indicate a significant difference between groups. Data were analyzed using one-way ANOVA, followed by Tukey’s post hoc test.

Significant differences in motor activity were observed on day 14. The open-field test showed no significant difference between the control and *M. oleifera* treated groups in total distance moved (TDM) (*p* *=* 0.9028) or velocity (*p* *=* 0.9934) (Fig. 6A,B). Reserpine administration significantly reduced TDM and velocity (*p* *<* 0.0001 for both) compared with controls, indicating impaired locomotor activity. However, *M. oleifera* treatment did not significantly improve TDM (*p =* 0.9762) or velocity (*p =* 0.9163) compared to the reserpine group. No significant differences were observed in rotarod performance on day 16 or forelimb grip strength on day 14 between the *M. oleifera*–treated group and the control group (*p* *=* 0.1793 and *p* *=* 0.0957, respectively). In contrast, reserpine administration significantly reduced rotarod latency to fall, with mice falling within the first few seconds, and markedly decreased forelimb grip strength, as evidenced by lower recorded gram force values compared with the control group (*p* *<* 0.0001 and *p* *=* 0.0125, respectively). Treatment with *M. oleifera* (500 mg/kg) significantly improved both rotarod performance and grip strength in the FM + *M. oleifera* group compared with the FM group (*p* *<* 0.0001 and *p =* 0.0001, respectively) (Fig. 6C,D).

### 3.5 The anti-depression protective effect of *M. oleifera* in FM mice model

**Fig. 7:**
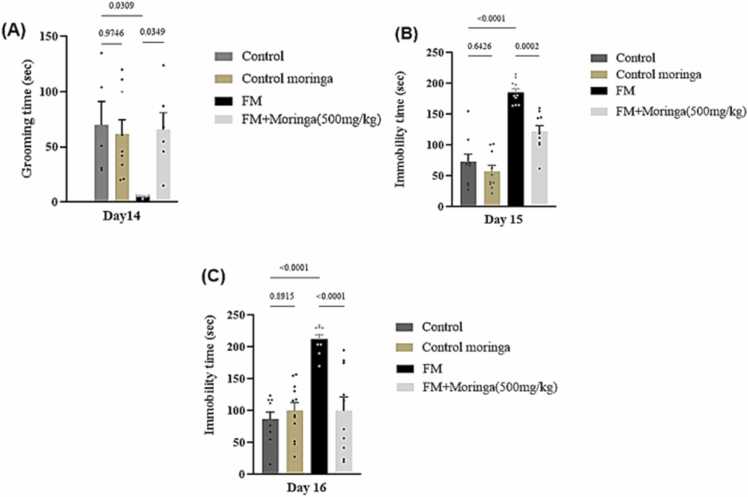
The effect of *M. oleifera* on depression like behavior in FM mice model induced by reserpine (A) Grooming time in ST; **(B)** Immobility time in TST at day 15; **(C)** Immobility time in FST at day 16. SEM present by the vertical lines for each group (n = 20 mice), and all bars show mean. Exact *p*-values are shown above each column to indicate a significant difference between groups. Data were analyzed using one-way ANOVA, followed by Tukey’s post hoc test.

The splash test on day 15 revealed no significant difference in grooming time between the control and *M. oleifera* groups (*p* *=* 0.9746) (Fig. 7A). Reserpine administration significantly reduced grooming time compared to the control (*p =* 0.0309), while the FM + *M. oleifera* group showed a significant improvement (*p* *=* 0.0349). In the tail suspension test (day 15), no significant difference in immobility time was observed between the control and *M. oleifera* groups (*p =* 0.6426) (Fig. 7B). Reserpine markedly increased immobility time compared to control (*p* *<* 0.0001), whereas FM + *M. oleifera* treatment significantly reduced it (*p* *=* 0.0002). Likewise, in the forced swimming test (day 16), immobility time did not differ between control and *M. oleifera* groups (*p =* 0.8915) (Fig. 7C). Reserpine significantly increased immobility duration (*p <* 0.0001), while FM + *M. oleifera* treatment significantly decreased it (*p* *<* 0.0001).

### 3.6 Protective role of *M. oleifera* against histology alterations induced by reserpine in hippocampus areas (Dentate gyrus and CA3 regions)

**Fig. 8:**
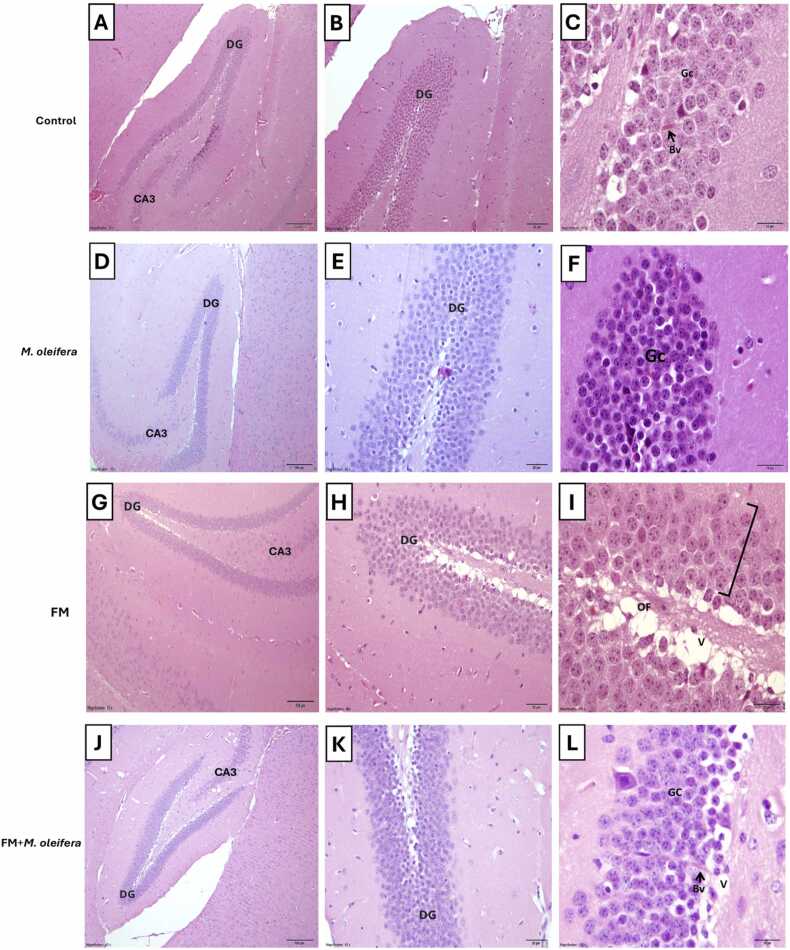
A photomicrograph of the hippocampus of male Swiss albino mice. (A, B,C) Control group appears normal major areas (CA3) and the dentate gyrus area (DG), showing of granular cells (Gc) arranged in layer that appear rounded in shape with large deep stain nucleus and normal blood vessels (Bv); (D,E,F) *M. oleifera* group appear of dentate gyrus, showing normal granular cells (Gc) arranged in layer that appear rounded in shape with large deep stain nucleus; (G,H,I) FM group appears of dentate gyrus region, granular cell layer (black bracket) appears disorganized, showing open face nuclei (OF) and appear vacuolated cell layer(V); (J,K,L) Fm+*M. oleifera* group appear of dentate gyrus region,granular cells (Gc) appear densely packed of a rounded less disorganized,appear few vacuoles(V) and normal blood vessels(Bv) (The scale bar 10 µm, stain H&E; Magnification = X10, X20, X40, X100).

**Fig. 9:**
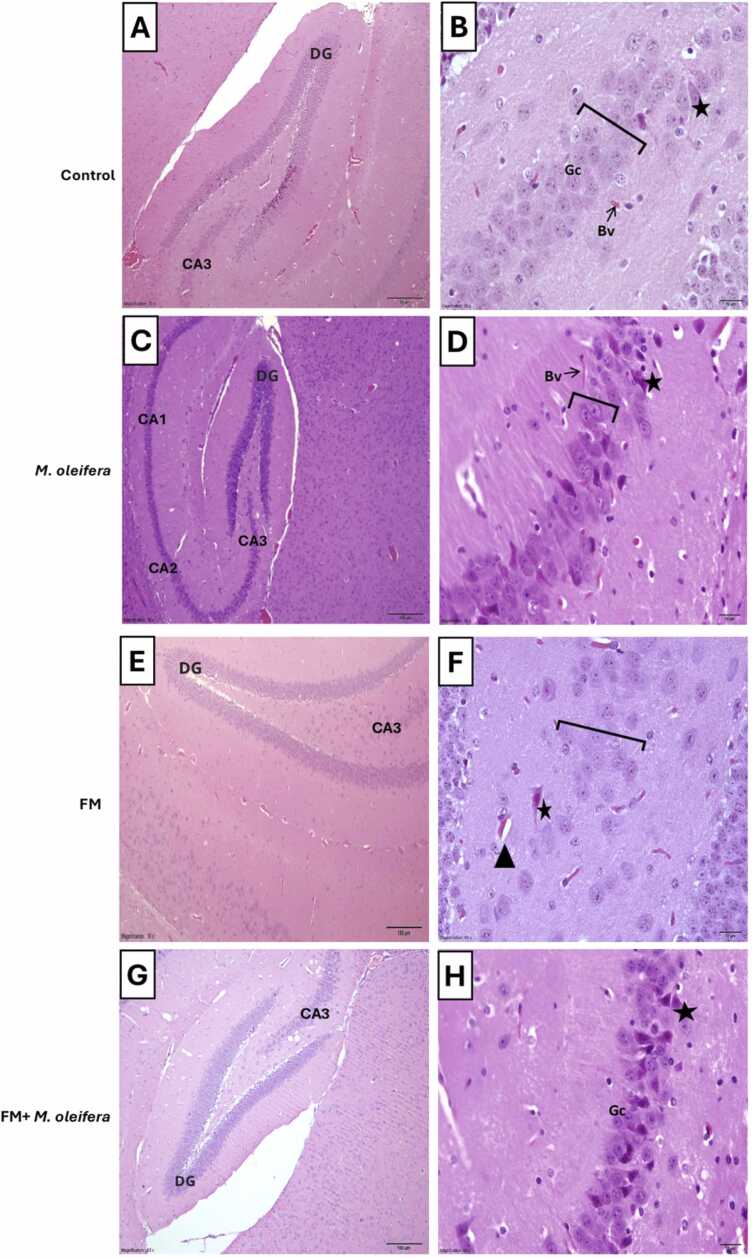
A photomicrograph of the hippocampus of male Swiss albino mice. (A,B) Control group appear normal(CA3) region and the dentate gyrus area (DG), granular cell (black bracket) (Gc) that appear rounded in shape with large deep stain nucleus, pyramidal cell (black star), and normal blood vessels (Bv); (C,D) *M. oleifera* group appear CA3 region granular cell (black bracket) appear rounded in shape with large deep stain nucleus and pyramidal cell (black star); (E,F) FM group showing of loss of normal architecture of granular cell and less of packed cells of CA3, appear dilation blood vessels (black triangle), notice loss of normal shape of pyramidal cell (black star); (G,H) FM+ *M. oleifera* group appear of (CA3) region, showing normal granular cell (GC) and normal shape of pyramidal cell(black star). (The scale bar 10 µm, stain H&E; Magnification = X10, X20, X60).

Histological examination of the control group revealed normal hippocampal architecture consists of major areas the cornu ammonis (CA3) and the dentate gyrus area (DG) (Fig. 8A,B). In dentate gyrus characterized by densely packed, well-organized granular cells (Gc) with rounded morphology and deeply stained nuclei and blood vessels (Bv) (Fig. 8C). The control group revealed normal hippocampal architecture consists of major areas such as the cornu ammonis (CA3) and the dentate gyrus area (DG) (Fig. 9 A). Moreover, the CA3 region displayed healthy, granular cell with large, deeply stained nuclei, normal pyramidal cell, and normal blood vessels (Fig. 9B). In the dentate gyrus (DG) region of the Moringa-treated group on day 17(Fig. 8D,E), the granular cell appeared normal, containing densely packed, rounded granular cell (Gc) (Fig. 8F). Normal hippocampal architecture consists of major areas such as the cornu ammonis (CA1, CA2, CA3) and the dentate gyrus area (DG) (Fig. 9 C), the CA3 region showed healthy granular cell, exhibiting rounded morphology with deeply stained nuclei and normal pyramidal cell (Fig. 9D).

Histological sections of the reserpine group showed disrupted dentate gyrus area (DG) architecture (Fig. 8 G,H), with a disorganized granular cell layer, open-faced nuclei (OF) and vacuolated areas (V) in the dentate gyrus (Fig. 8I). The CA3 region (Fig. 9E), exhibited a loosely granular cell, distorted pyramidal cell, and dilated blood vessels (Fig. 9 F).

In the FM + Moringa group, the dentate gyrus (DG) region (Fig. 8J, K). Dentate gyrus (DG) region displayed densely packed, rounded granular cells (Gc) with minimal disorganization and few vacuoles (V) and blood vessels (Bv) (Fig. 8L). The CA3 region (Fig. 9 G), exhibited restored granular cell showing rounded morphology and deeply stained nuclei, as well as mostly normal pyramidal cell (Fig. 9H).

### 3.7 Protective effect of *M. oleifera* against histology alterations induced by reserpine in Thalamus of FM mice model

**Fig. 10:**
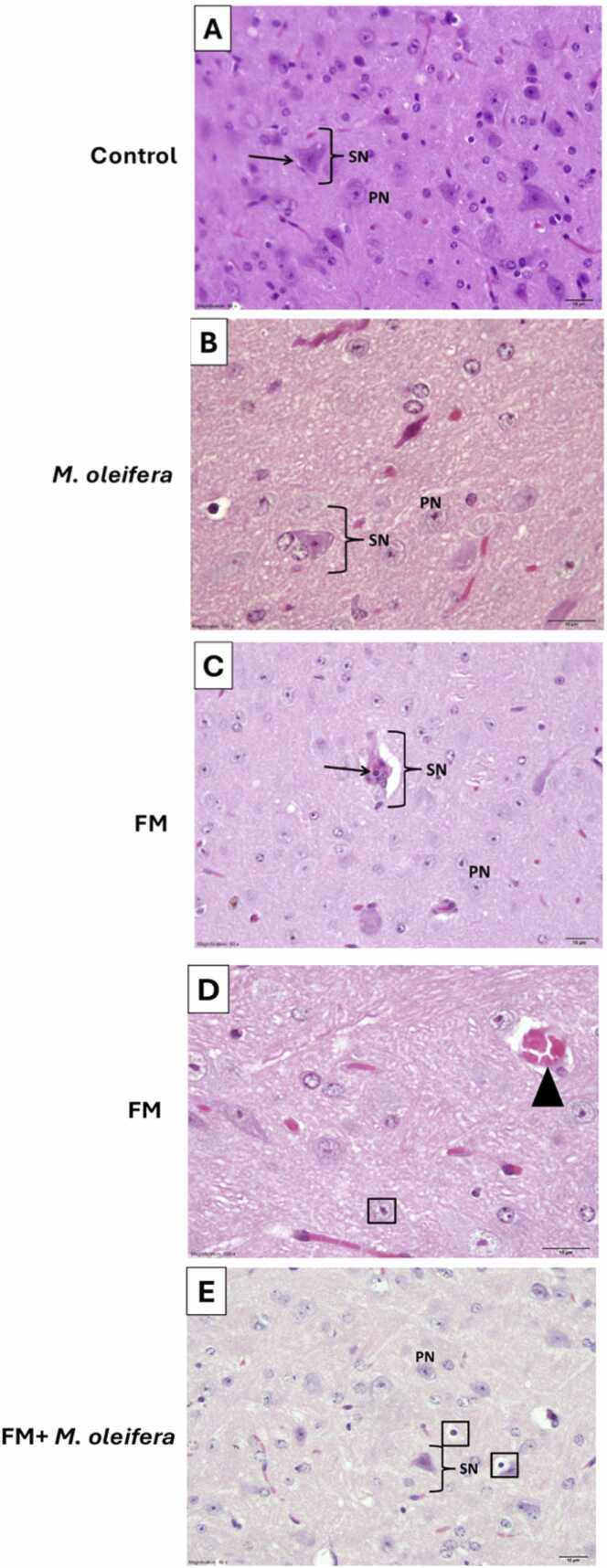
A photomicrograph of the thalamus of male Swiss albino mice. (A) represents control group noticed normal healthy sensory neuron ((SN) black arch) appear with pyramidal shaped cell body and prominent nucleus (black arrow) and normal thalamic principal cell (PN); (B) represents moringa treated group with normal distribution of the thalamic principal cell (PN), noticed normal healthy sensory neuron appears with pyramidal shaped cell body and prominent nucleus (black arch (SN)); (C&D) represent FM group showing decrease density of principle thalamic cells (PN) with unhealthy sensory neuron ((SN) black arch) apparently binucleated cell (thick arrow)and principle thalamic cells accidentally with pyknotic nuclei (inside a box). Notice marked dilation in blood vessels with hemorrhagic area (black triangle); and (E) represents FM+ *M. oleifera* group showing normal of principal thalamic cells (PN), with accidentally pyknotic nuclei (inside a box), normal sensory neuron pyramidal shaped cell body with prominent nucleus ((SN) black arch)). (A&C: the scale bar 10 µm, H&E stain; X 60; B,D&E: The scale bar 10 µm, H&E stain; X 100).

The thalamus of the control group exhibited normal histoarchitecture, showing well-distributed thalamic principal cells (PN) and healthy sensory neurons (SN) with pyramidal-shaped cell bodies and prominent nuclei (Fig. 10A). Similarly, the Moringa-treated group showed normal thalamic structure with abundant principal cells and intact sensory neurons exhibiting typical pyramidal morphology (Fig. 10B).

The thalamic region of the FM group showed decreased density of principal thalamic cells (PN) and degenerated sensory neurons (SN), some appearing binucleated (Fig. 10C). Principal cells exhibited pyknotic nuclei with markedly dilated blood vessels and focal hemorrhagic areas (Fig. 10D). In contrast, the FM + Moringa group showed largely normal thalamic architecture, with well-preserved principal cells, occasional pyknotic nuclei, and normal sensory neurons with prominent nuclei (Fig. 10E).

### 3.8 Role of *M. oleifera* on IL-1β, TNF-α and NO levels in FM mice model

**Fig. 11:**
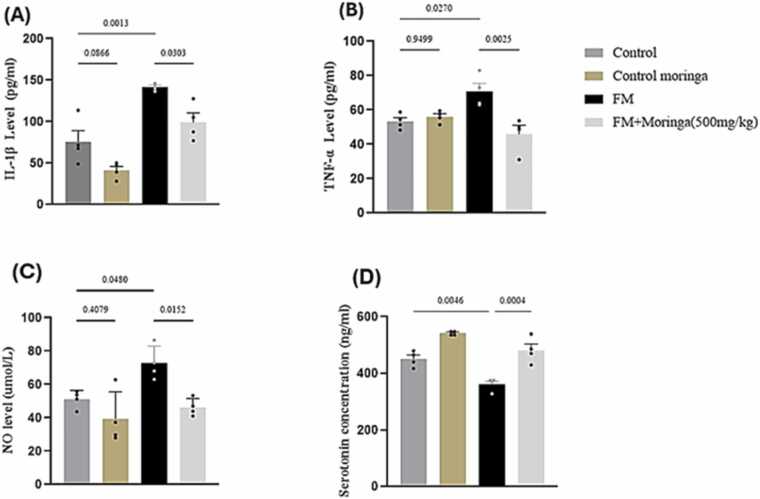
Effect of *M. oleifera* on IL-1β (A), TNF-α (B), NO (C) and serotonin levels (D) in brain tissues of FM mice model. Data was present as Mean±SEM for each group (n = 20 mice). Exact *p*-values are shown above each column to indicate a significant difference between groups. Data were analyzed using a one-way ANOVA and Tukey's test was applied.

No significant differences were observed between the control and *M. oleifera* groups in Interleukin-1β (IL-1β), Tumor Necrosis Factor-α (TNF-α), or Nitric Oxide (NO) levels (Fig. 11A–C). In contrast, reserpine treatment markedly elevated IL-1β, TNF-α, and NO levels compared to the control group (*p* *=* 0.0013, *p* *=* 0.0270, *p* *=* 0.0480, respectively). The FM + *M. oleifera* group showed significant improvement, with reduced levels of these inflammatory markers relative to the reserpine group (*p* *=* 0.0303, *p* *=* 0.0025, *p* *=* 0.0152, respectively). (Fig. 11A–C).

Serotonin concentration was significantly elevated in the *M. oleifera*-treated group compared to the control (Fig. 11D). In contrast, the reserpine group showed a significant reduction in serotonin levels (*p =* 0.0046). Treatment with FM + *M. oleifera* markedly increased serotonin concentration compared to the FM group (*p* *=* 0.0004) (Fig. 11D).

## 4 Discussion

The underlying causes of fibromyalgia (FM) remain poorly understood; consequently, the disorder continues to attract considerable scientific attention due to its complex clinical manifestations, particularly chronic pain and depression. Moreover, the pathophysiology of FM has not yet been fully elucidated, and no effective or officially approved treatment is currently available (Singh et al., 2021). Although conventional therapies, such as antidepressants, are widely used for the management of chronic pain, their associated adverse effects may worsen the condition. Therefore, the present study aimed to investigate the potential of natural compounds in alleviating FM symptoms. Specifically, this study examined the protective effects of *M. oleifera* against reserpine-induced FM in a mouse model through behavioral assessments, histopathological evaluations, and biochemical analyses of FM-related markers.

The present findings demonstrated that reserpine treatment induced a significant reduction in body weight, in agreement with previous reports (Nagakura et al., 2018). Reserpine is known to cause depression and fatigue by depleting neurotransmitters such as serotonin, which subsequently reduces food intake and leads to weight loss (Harris et al., 1998). However, on days 14 and 17 of the experimental period, administration of *M. oleifera* extract following reserpine treatment resulted in a significant increase in body weight. This effect may be attributed to the ability of *M. oleifera* to restore neurotransmitter balance and enhance locomotor activity. These observations are consistent with the findings of Ray et al. (2003), who reported that pretreatment of penicillin-induced seizure rats with *M. oleifera* extract significantly modulated locomotor behavior.

The present study is the first to demonstrate the protective effects of *M. oleifera* in a fibromyalgia (FM) model. Reserpine administration induced the development of mechanical allodynia, as indicated by a reduced paw withdrawal threshold in the von Frey test, along with decreased latency in the hot-plate test, reflecting increased sensitivity to thermal pain. In line with these observations, Giacoppo et al. (2017) reported that moringin, an isothiocyanate compound derived from *M. oleifera*, exhibited therapeutic potential in experimental autoimmune myelitis; topical application of moringin cream for 21 days significantly improved disease outcomes in treated mice.

In this study, the effect of *M. oleifera* leaf extract on pain was evaluated using the hot-plate and von Frey tests, despite the lack of prior FM-specific research on *M. oleifera*. The results demonstrated that *M. oleifera* significantly increased the paw withdrawal threshold, indicating notable analgesic activity. These findings align with those of Vafaeian et al. (2025), who reported that *M. oleifera* extract effectively reduced pain in rats in both tests. The observed increase in latency is also consistent with previous reports (Bhattacharya et al., 2014). The analgesic effect of *M. oleifera* may involve activation of central opioid pathways through the release of endogenous peptides in the periaqueductal gray matter, while its flavonoid constituents may further enhance pain relief by inhibiting prostaglandin synthesis (Bhattacharya et al., 2014).

Reserpine depletes dopamine, serotonin, and norepinephrine, resulting in reduced motor activity, muscle weakness, and heightened pain sensitivity hallmarks of fibromyalgia-like symptoms (Yao et al., 2020). In the present study, behavioral assessments revealed that reserpine-treated mice exhibited diminished muscle strength and motor performance. In the open-field test, these mice showed prolonged immobility compared to the control and treatment groups, aligning with previous findings (Ferrarini et al., 2022). Similarly, the significant reduction in grip strength and shorter latency in the rotarod test further confirm the role of reserpine-induced neurotransmitter depletion in motor impairment (Yao et al., 2020, Ferrarini et al., 2022).

Numerous natural products have been evaluated in the reserpine-induced fibromyalgia model, which reproduces monoaminergic depletion, oxidative stress, neuroinflammation, and pain hypersensitivity. Polyphenolic compounds such as curcumin, resveratrol, quercetin, and green tea catechins primarily exert protective effects by reducing lipid peroxidation and restoring antioxidant defenses, resulting in partial attenuation of hyperalgesia and depressive-like behaviors (Hussain et al., 2016, Shen et al., 2022). Adaptogenic plants, including Withania somnifera and Panax ginseng, improve stress resilience and neurotransmitter imbalance, mainly through antioxidant and mitochondrial-supportive mechanisms (Kim et al., 2017, Paul et al., 2021). However, most tested natural agents act on limited molecular targets. In contrast, *M. oleifera* possesses a distinctive phytochemical composition that enables concurrent modulation of oxidative stress, neuroinflammation, mitochondrial dysfunction, and monoaminergic signaling, supporting a multitarget, systems-level mechanism that may offer broader therapeutic relevance in fibromyalgia.

Moreover, *M. oleifera* possesses a distinctive phytochemical profile rich in bioactive isothiocyanates (e.g., moringin), flavonoids (quercetin, kaempferol), and phenolic acids, which collectively exert multitarget actions relevant to FM pathophysiology. Unlike many natural extracts that primarily modulate oxidative stress, *M. oleifera* has been shown to simultaneously activate the Nrf2/ARE antioxidant pathway while suppressing NF-κB–mediated neuroinflammatory signaling, providing coordinated redox–inflammatory regulation (Jaja-Chimedza et al., 2017, Kou et al., 2018). This dual mechanism is particularly relevant to the reserpine model, which involves oxidative imbalance, neuroinflammation, and monoaminergic depletion. Through the restoration of redox homeostasis and mitigation of neuroinflammation, *M. oleifera* may preserve neuronal integrity and dampen central sensitization, ultimately leading to the observed improvement in nociceptive and behavioral outcomes. These findings support the potential of *M. oleifera* as a neuroprotective and analgesic agent in fibromyalgia-like.

The antioxidant and anti-inflammatory properties of *M. oleifera* contribute to the enhancement of movement-related neurotransmitters, thereby improving neuromotor function and neuromuscular coordination (Alqahtani and Albasher, 2021). Razzaq et al. (2020) reported significant improvements in grip strength and rotarod performance following *M. oleifera* administration. However, in the open-field test, which evaluates exploratory and general motor behavior, *M. oleifera* did not produce a significant improvement (Mahmoud et al., 2017). These findings suggest that noticeable enhancement in complex behaviors, such as exploration, may require prolonged treatment duration or higher extract doses (Castaño Barrios et al., 2021).

Depression and anxiety are key symptoms associated with fibromyalgia (FM) (Fusco et al., 2019). Reserpine has been shown to deplete monoamines, including dopamine and serotonin, in the central nervous system, which play crucial roles in mood regulation and modulating pain and fear responses (Peres et al., 2016, Singh et al., 2021). In the present study, three behavioral tests were employed to assess depressive-like behaviors. In the splash test, reserpine-treated mice exhibited reduced grooming and activity compared to controls, indicating diminished self-care behavior. Similarly, in the tail suspension test, reserpine induced depression-like responses and impaired social interaction (Martins, et al., 2022). In the forced swim test, mice showed prolonged immobility and reduced swimming activity, consistent with the depression-like phenotype reported by Brusco et al. (2019) and Singh et al. (2021).

One of the primary objectives of this study was to evaluate the efficacy of the methanolic extract of *M. oleifera* leaves in alleviating depression-like symptoms associated with FM, using established behavioral models of depression (Jaiswal et al., 2009). *M. oleifera* possesses notable medicinal properties attributed to its antioxidant constituents, particularly flavonoids, which contribute to its anti-inflammatory and neuroprotective effects. The present findings revealed that *M. oleifera* treatment significantly reduced immobility time in both the tail suspension and forced swim tests, accompanied by an increase in swimming duration. Furthermore, treated mice exhibited enhanced grooming behavior in the splash test, indicating improved motivational and self-care activity. These results are in line with the findings of Kaur et al. (2015), who reported that *M. oleifera* extract ameliorates depressive-like behaviors through its antioxidant and anti-inflammatory mechanisms.

The present study demonstrated impaired spatial memory in FM–induced mice following reserpine administration. This was evident in the Y-maze test, where a significant reduction in the percentage of correct alternations indicated cognitive decline. These findings align with previous reports linking elevated reactive oxygen species (ROS) and reduced antioxidant defenses, such as glutathione, to neuronal damage in memory-associated brain regions like the hippocampus (Martins et al., 2022, Park and Jeong, 2024). Moreover, treatment with *M. oleifera* markedly improved spatial memory, likely through its anti-inflammatory and antioxidant effects, which protect hippocampal neurons from oxidative injury, consistent with findings from a mouse model of memory impairment (Arozal et al., 2022).

The hippocampus is essential for memory, learning, and pain regulation and is highly affected by neuroinflammation, oxidative stress, and neurotransmitter imbalance (Wood, 2004). This study assessed the protective effects of *M. oleifera* against reserpine-induced histological alterations in the brain. Reserpine-treated mice showed marked hippocampal damage, including loss of normal dentate gyrus architecture, degeneration of the granule cell layer, ruptured pyramidal neurons, vacuolation, vascular dilation, and reduced cell density in the CA3 region. These findings are consistent with AboTaleb et al. (2024), who reported similar reserpine-induced histopathological changes in the hippocampus.

Clinical studies have reported a reduction in hippocampal volume in patients with fibromyalgia, suggesting that neurological alterations may contribute to the disease’s symptoms. This observation aligns with the present findings in the FM mouse model (Leon-Llamas et al., 2021). Several studies have similarly demonstrated that reserpine administration depletes neurotransmitters, leading to a reduction in CA3 region volume (Peres et al., 2016, Li et al., 2023). Such neuronal alterations may underlie the development of depression and mood disturbances associated with fibromyalgia (Peres et al., 2016). Therefore, alleviating neuroinflammation in the hippocampus may contribute to protection against reserpine-induced neurodegeneration (Li et al., 2023). Consistent with this, the present study demonstrated that 17 days of *M. oleifera* supplementation markedly reduced neuroinflammation and restored dentate gyrus and CA3 region integrity, showing well-organized granule cell layers and normal pyramidal neurons. Likewise, Onasanwo et al. (2021) reported that a diet enriched with *M. oleifera* exerts neuroprotective effects and may serve as a potential therapeutic or pharmacological agent for preventing neuronal loss.

The underlying cause of pain in the FM mouse model remains poorly understood. However, several studies suggest that neurotransmitter imbalance within the central nervous system contributes to heightened pain sensitivity (Staud, 2006). Singh et al. (2021) reported that FM is associated with reduced levels of biogenic amines, particularly serotonin, which plays a crucial role in descending pain-modulating pathways of the brain and spinal cord by activating endogenous analgesic mechanisms. Low serotonin levels are therefore linked to increased pain perception in FM Singh et al. (2021). In the present study, reserpine administration significantly reduced serotonin levels compared with controls, consistent with its known inhibition of the vesicular monoamine transporter-2 (VMAT-2), leading to depletion of brain biogenic amines (Sousa et al., 2018). Conversely, treatment with *M. oleifera* extract markedly increased serotonin levels, supporting the findings of Ray et al. (2003), who demonstrated that aqueous *M. oleifera* root extract elevated serotonin and reduced norepinephrine and dopamine levels in a penicillin-induced convulsion model, thereby exerting neuroprotective and antiepileptic effects. Moreover, El-Kadi et al. (2025) proved that *M. oleifera* extract has been reported to restore impaired 5-HT1A receptor signaling and normalize hippocampal serotonin (5-HT) levels in rats subjected to chronic unpredictable mild stress (CUMS), an effect that is associated with its notable anxiolytic and antidepressant-like properties.

The present study demonstrated elevated oxidative enzyme levels in the FM mouse model induced by reserpine. FM is characterized by increased activity of monoamine oxidase A (MAO-A) in the brain, which promotes neurotransmitter degradation Singh et al. (2021). This imbalance accelerates oxidative processes by enhancing reactive oxygen species (ROS) production and reducing antioxidant defenses, resulting in neuronal damage, particularly in the hippocampus and hypothalamus Singh et al. (2021). Additionally, several studies have reported elevated levels of inflammatory cytokines such as IL-1β and TNF-α in FM, which stimulate prostaglandin and substance P production, leading to glial activation, neuronal injury, pain, and memory impairment (Golan et al., 2004, Zhang and An, 2007). Consistent with these findings, the current results revealed increased brain levels of IL-1β and TNF-α in reserpine-treated mice. Furthermore, elevated oxidative stress and nitric oxide (NO) levels have been implicated in FM pathophysiology and symptom aggravation (Fusco, et al., 2019). In this study, reserpine treatment significantly increased brain NO levels, supporting the strong interconnection between oxidative stress and inflammation, where oxidative damage induces inflammatory responses, and sustained inflammation further amplifies oxidative injury (Fais et al., 2017).

The current work proved a significant reduction in the inflammatory cytokines IL-1β and TNF-α in the *M. oleifera* treated group, supporting its role in modulating the excessive inflammatory response associated with chronic pain. These findings are consistent with Alqahtani and Albasher (2021), who reported that *M. oleifera* extract suppressed IL-1β and TNF-α elevation in the cerebral cortex. Additionally, oral administration of *M. oleifera* significantly decreased brain NO levels, in agreement with previous studies (Alqahtani and Albasher, 2021). Neurodegeneration is known to upregulate NF-κB p65, a key transcription factor promoting apoptotic and inflammatory mediators such as TNF-α, IL-1β, and iNOS (Alqahtani and Albasher, 2021). In the present study, *M. oleifera* treatment reduced IL-1β, TNF-α, and NO levels, suggesting that its anti-inflammatory effect may involve inhibition of NF-κB p65 activation. Additionally, studies employing rat models of chronic stress and inflammation have demonstrated that *Moringa oleifera* extract modulates serotonin levels while attenuating the production of pro-inflammatory cytokines. These observations support a potential therapeutic role for *M. oleifera* in disorders characterized by neuroinflammation and neurotransmitter imbalance, including fibromyalgia (de Siqueira Patriota et al., 2025). Collectively, these findings indicate that *M. oleifera* exerts a protective effect against reserpine-induced fibromyalgia by suppressing NF-κB–mediated cytokine production and mitigating neuroinflammation.

The protective effects of *M. oleifera* observed in the reserpine-induced fibromyalgia mice model may have potential clinical relevance, as oxidative stress and neuroinflammation are key contributors to fibromyalgia pathophysiology in humans (Häuser et al., 2015). By mitigating oxidative damage, restoring redox balance, and reducing neuroinflammatory mediators, *M. oleifera* could theoretically improve pain perception, fatigue, and cognitive dysfunction in fibromyalgia patients. Moreover, its bioactive constituents, including polyphenols, flavonoids, and isothiocyanates, are generally considered safe and have been evaluated in clinical trials for other chronic inflammatory and oxidative stress-related conditions (Jaja-Chimedza et al., 2017, Kou et al., 2018). However, several limitations should be acknowledged. First, the current findings are based on an animal model, and the reserpine-induced fibromyalgia phenotype may not fully recapitulate the complex, multifactorial nature of human fibromyalgia. Second, the pharmacokinetics, bioavailability, and optimal dosing of *M. oleifera* extracts in humans remain unclear. Finally, long-term safety and potential interactions with standard fibromyalgia therapies require further investigation. Therefore, while the present results provide mechanistic insights and preclinical support for the therapeutic potential of *M. oleifera*, clinical studies are essential to determine its efficacy and safety in patients.

## 5 Conclusions

The present findings suggest that the protective effects of *M. oleifera* extract against reserpine-induced fibromyalgia may be attributed to modulation of immune responses and inhibition of inflammatory cytokine signaling pathways. These results provide experimental support for the traditional use of *M. oleifera* as a natural therapeutic agent in managing inflammation-associated disorders. However, in the absence of clinical evidence, the findings should be considered preliminary and hypothesis-generating, serving as a basis for future mechanistic and clinical studies.

## CRediT authorship contribution statement

**Badrah S. Alghamdi:** Software, Resources, Formal analysis, Data curation. **Alhawiti Ohoud Hamdan:** Writing – review & editing, Writing – original draft, Methodology, Investigation, Formal analysis, Data curation, Conceptualization. **Batawi Ashwaq:** Writing – review & editing, Supervision, Investigation, Funding acquisition, Data curation, Conceptualization. **Sheren A. Azhari:** Visualization, Software, Methodology, Data curation. **Asma Almuhammadi:** Methodology, Investigation, Formal analysis. **Ashwaq Hassan Alsabban:** Writing – review & editing, Methodology, Investigation. **Mona A. AL-Thepyani:** Methodology, Investigation. **Reham Tash:** Methodology, Investigation. **Thepyani Mona A. AL:** Writing – original draft, Software, Resources, Methodology. **Ashwaq Hassan Alsabban:** Writing – review & editing, Visualization, Supervision, Formal analysis. **Sheren A. Azhari:** Supervision, Investigation, Formal analysis, Data curation, Conceptualization. **Ohoud H. Alhawiti:** Writing – original draft, Methodology, Investigation, Data curation, Conceptualization.

## Ethical statement

All animal experimental protocols were performed under the approval of the Animal Care and Use Committee of King Abdulaziz University (Ethical Approval No. 358–24).

## Funding

This research was funded by 10.13039/501100004054King Abdulaziz University, Jeddah, Saudi Arabia, through the Ph.D. program.

## Declaration of Competing Interest

The authors declare that they have no known competing financial interests or personal relationships that could have appeared to influence the work reported in this paper.

## Data Availability

Data supporting the findings of this study are available from the **c**orresponding authors upon reasonable request.
